# Postpartum Posterior Reversible Encephalopathy Syndrome (PRES): Three Case Reports and Literature Review

**DOI:** 10.1155/2019/9527632

**Published:** 2019-01-27

**Authors:** Eleonora Marcoccia, Maria Grazia Piccioni, Michele Carlo Schiavi, Vanessa Colagiovanni, Ilaria Zannini, Angela Musella, Virginia Sibilla Visentin, Flaminia Vena, Gabriele Masselli, Marco Monti, Giuseppina Perrone, Pierluigi Benedetti Panici, Roberto Brunelli

**Affiliations:** ^1^Department of Experimental Medicine, Policlinico Umberto I, Sapienza University of Rome, Viale del Policlinico 155, Rome, Italy; ^2^Department of Gynecology, Obstetrics and Urological Sciences, Policlinico Umberto I, Sapienza University of Rome, Viale del Policlinico 155, Rome, Italy; ^3^Department of Obstetrics and Gynecology, “E. De Santis Hospital”, Via Achille Grandi, Genzano, Italy; ^4^Department of Radiology, Policlinico Umberto I, Sapienza University of Rome, Viale del Policlinico 155, Rome, Italy

## Abstract

Posterior reversible encephalopathy syndrome is a rare complication generally associated with headache and acute changes in blood pressure. Delay in the diagnosis and treatment may result in death or in irreversible neurological sequelae. We present three cases of PRES occurring in young women during puerperium. We report a literature review ranged from January 1990 to June 2015 describing clinical features, diagnostic and medical approach, and maternal outcome.

## 1. Introduction

Posterior reversible encephalopathy syndrome (PRES) is a rare complication in patients with acute hypertensive disorders. It is also described as a complication after chemotherapy, infection, sepsis, autoimmune diseases, and hypercalcemia (cytotoxic edema) [1]. It was described first by Hinchey et al. in 1996 [1]. This syndrome is manifested by neurologic symptoms: headache, nausea or vomiting, generalized seizures, visual disturbance, and altered sensorium whereby the vasogenic edema of the subcortical white matter occurrs in the posterior occipital and parietal lobes [2]. Recurrent seizures are common and visual disturbances are present ranging from hemianopsia and visual neglect to cortical blindness [3]. Pathogenesis of PRES remains unclear but it seems to be associated with vasogenic edema in occipital lobe. Preeclampsia/HELLP syndrome, immunosuppressive/cytotoxic drugs, thrombotic thrombocytopenic purpura/hemolytic uremic syndrome, acute or chronic renal diseases, steroid therapy, and liver failure seem to be the causes of the onset of edema [4]. Clinical suspects of PRES have to be confirmed by Magnetic Resonance Imaging (MRI). The most characteristic imaging pattern in PRES is the presence of edema involving the white matter of the posterior portions of both cerebral hemispheres, especially the parietooccipital regions [5]. Narbone et al. suggest defining this condition as potentially RES, to emphasise that the posterior localization of the lesions, even if constant, may not represent the most relevant finding in some patients and that reversibility is not spontaneous but is usually related to an adequate treatment [6]. An early diagnosis is primary in order to start therapy and avoid mortality and morbidity in terms of long and short temp complications.

We present three cases of PRES occurring in young women, during puerperium. We then performed a literature review regarding cases of PRES in puerperium reported from January 1990 to June 2015.

## 2. Cases Presentation

### 2.1. Case n° 1

A 21-year-old woman primigravida with no previous history of hypertension or other risk factors for PRES underwent a Cesarean section (CS) at the 39th week for breech presentation. From postoperative day 1, she developed hypertension (170/105 mmHg), associated with cefalea and periorbital edema. Hypertension was treated with clonidine hydrochloride 0.15 mg/kg. Despite therapy on the postoperative day 7 blood pressure increased (180/115 mmHg) without proteinuria. Parenteral magnesium sulfate (4g 20/min IV and 1-2 g/h infusion) was started. The patient developed severe headache and generalized tonic-clonic seizure treated with Diazepam iv 10 mg. After the seizure the patient showed alertness, mydriasis, and decrease in visual acuity. The same day computed tomography (CT) showed focal areas of hypodensity in the right hemisphere and hyperdensity at the right cerebellar pontine angle. Then a cerebral MRI was performed and axial FLAIR MRI demonstrated bioccipital foci of high signal intensity involving the cortex and subcortical white matter with normal Diffusion Weighted Images (DWI).

**Figure 1:**
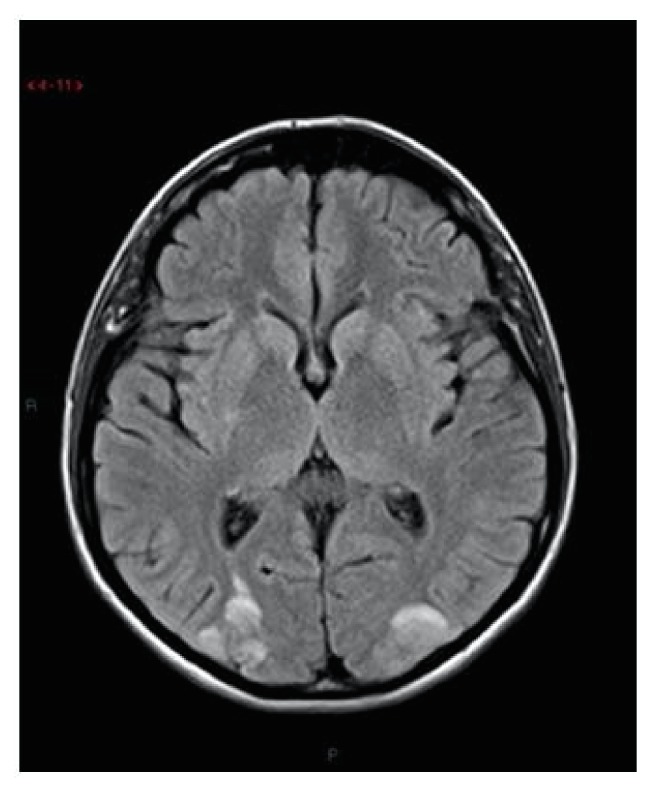
Axial FLAIR magnetic resonance images demonstrated bioccipital foci of high signal intensity involving the cortex and subcortical white matter.

**Figure 2:**
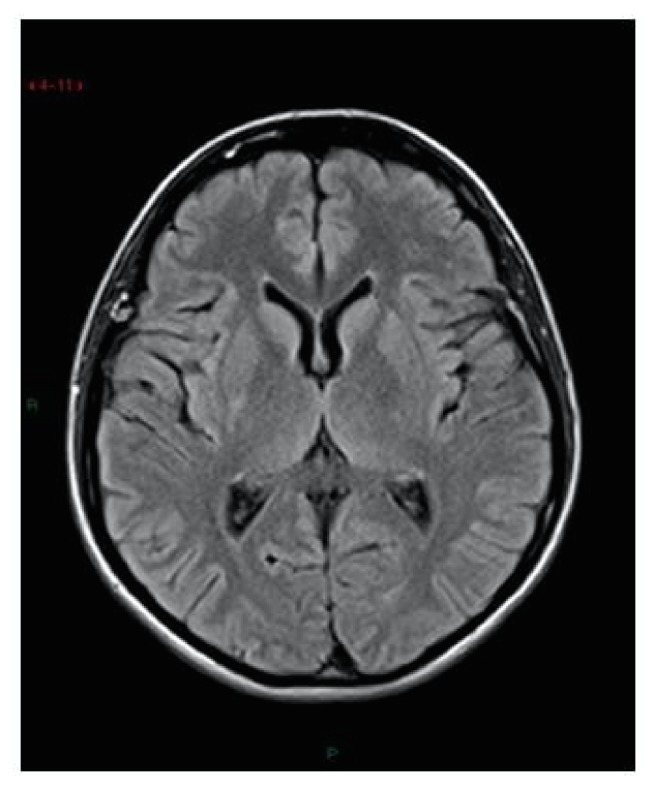
Magnetic resonance images performed 7 days after the first examination. There are no signs of foci of high signal intensity.

These findings were indicative of vasogenic edema due to cerebrovascular autoregulatory dysfunction, according to PRES. In the same day the patient developed another generalized tonic-clonic seizure, treated with Diazepam iv 10 mg, and bilateral blindness. A neurological consultation was requested, but no focal neurologic signs were detected. EEG showed a frontal-occipital focal epileptogenic localization. Blood gas analysis showed a severe acidosis (pH: 7.26; BE: -10,5). The patient was treated with Phenytoin 50mg, Mannitol 100mg x 4 t.i.d. (ter in die), and bicarbonate to correct acidosis. On day postoperative 10, blood pressure was stable and the patient was in better clinical condition, with improved vision. Radiological findings resolved on MRI performed at 7 days after the first examination (Figures 1 and 2). Moreover, periodic electroencephalogram, TransCranial EcoColorDoppler, and one ophthalmological screen to value any permanent damage were performed. Two months after Transcranial Eco ColorDoppler still revealed increased Posterior Cerebral Artery Velocimetry. Two months later there was a full normalization of EEG and Transcranial EcoColorDoppler parameters and the patient suspended any therapy. No ophthalmological and neurological permanent damage persisted after 1-year follow-up.

### 2.2. Case n° 2

A 29-year-old woman primigravida with no previous history of hypertension or other risk factors for PRES was admitted to our department at 40/3 weeks of gestational age because of preterm rupture of membranes. Blood pressure was normal at admission and there were no alteration in serologic examination. She delivered after labour induction with oxytocin the day after admission. Intrapartum epidural was required by the patient and performed by an obstetric dedicated anesthetist with agreement of senior gynecologist. The patient developed severe headache in early puerperium. Bed rest in supine decubitus and intravenous therapy with fluid and Paracetamol (1 gr t.i.d.) was started in the suspect of postepidural cephalea. In postpartum day 6 she had an improvement in symptoms, but suddenly in postpartum day 7 she developed hypertension and a generalized tonic-clonic seizure treated with Diazepam iv 10 mg. After seizure the patient underwent to neuroprophylaxis with magnesium sulfate, a close anesthesiologic monitoring, and to a cerebral MRI. Axial and FLAIR MRI demonstrated cerebellar and occipital foci of high signal intensity involving the cortex and subcortical white matter with normal Diffusion Weighted Images (DWI), especially in the right hemisphere. Furthermore, an increased leptomeningeal enhancement was found; thus PRES was neuroradiologically diagnosed. EEG revealed a left holohemispheric epileptiform activity. The patient was admitted to Intensive Care Unit and treated with phenytoin urapidil and alfametildopa. Serum examinations were normal, excepted for an isolated increasing of LDH: 876 U/L.

Radiological findings resolved on MRI performed at 5 days after the first examination and LDH returned into normal values in 7 days after increasing.

### 2.3. Case n° 3

A gravida 1 para 0, 43-year-old woman, at 37 weeks gestation, was admitted to our clinic due to gestational hypertension. At the time of admission to hospital, her blood pressure was 140/90 mmHg and laboratory tests were normal, except ATIII 56% that was treated with infusion of 2000 UI of ATIII. There was no past history of hypertension nor other diseases except Gilbert's syndrome. The current pregnancy was physiological. The gestational hypertension was treated with methyldopa 250 mgx2. During the third day of recovery the woman started complaining of headaches and severe epigastric pain, and we administered corticosteroids (CS).

Five hours after delivery, the headaches rapidly increased in intensity, and the patient developed generalized tonic-clonic seizure. In the postictal state the patient showed alertness, mydriasis, and decrease in visual acuity. Blood pressure was 169/110–187/109 mmHg. With the anesthetists' recommendation, the woman was transferred to the Intensive Care Unit (ICU) for monitoring and management of seizure. At the admission in ICU the patient developed another generalized tonic-clonic seizure, treated with Diazepam iv 10 mg. I.v. MgSO4 was immediately administered, beginning with a loading dose of 4 g in 20 min, followed by a maintenance dose (i.v. 1 g per hour). Vital parameters were monitored every 15 min. ECG registered a sinus rhythm at 86 bpm. Laboratory tests reported increased liver enzymes (AST = 222 U/L, ALT = 170 U/L, CPK = 266 U/L, and LDH = 678 U/L) and a reduction in platelet count to 56 x 10^9^/L; serum bilirubin was 2,8 g/l, ATIII 47, and albumin 2,2 g/dL. Renal function tests, haematocrit level, and electrolytes were within normal limits. Findings were suggestive of postpartum preeclampsia complicated by HELLP syndrome [7]. Dexamethasone was promptly administered. Despite antihypertensive medications the patient continued complaining an occipital headache, as well as visual disturbances such as blurred vision. Due to the persistent headache and the decreased patient's alertness, brain MR was performed. The brain MR-imaging and MR-angiography of the circle of Willis were performed which showed cortical and subcortical hyperintense lesions in both cerebellar lobes with elevated diffusion and no angiopathy, imaging features related to vasogenic edema consistent with PRES syndrome (Figure 1) [7]. Neurological examination showed a drowsy patient in a confused state. Antiedemigenic agents (dexamethasone) and diuretic agent (furosemide) were administrated in addition to MgSO4 infusion; we witnessed a progressive state of consciousness improvement with neurological deficit resolution, biochemical analysis, and blood pressure normalization. EEG revealed an intense epileptogenic activity in the occipital lobe. The patient remained for 6 days in the ICU; then she came back to the Obstetric Department and on the 20th day after delivery she was discharged home without any symptom. The follow-up brain MRI performed 3 weeks later showed the complete resolution of brain oedema and no vascular imaging of abnormalities. The resolution further supported the diagnosis of PRES [7]. No neurological permanent damage persists after 1-year follow-up.

## 3. Materials and Methods

A research involving PubMed, EMBASE, Medline, and reference lists to identify articles published from January 1990 through June 2015 regarding PRES during puerperium was performed. The search was performed using “PRES in puerperium” as keywords, then in a second step we used the keywords “PRES in post-partum” in order to detect publications that eluded the first step of research. Our criteria for including reports in our analysis were development of PRES during puerperium, description of radiological diagnosis and therapies, and maternal outcome. Exclusion criteria were omitting at least one inclusion criteria. Maternal characteristics and clinical data were extracted. We then analyzed the timing of onset of PRES, instrumental diagnosis, drug therapy, patient outcome, and clinical and instrumental follow-up for each patient.

## 4. Results

**Figure 3:**
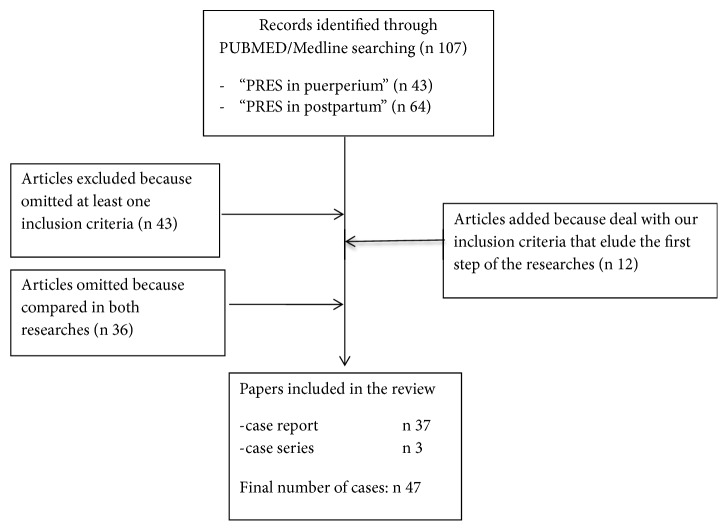
PRISMA 2009 flow diagram.

Our preliminary literature search identified 43 publications. When we used the keywords “PRES in post-partum” we obtained 64 results. We analyzed in a preliminary step 107 manuscripts. Seventy-nine articles were excluded from the review: 36 manuscripts because of being compared in both researches and other 43 because of omitting at least one inclusion criterion. We added to our analysis 12 further articles that had eluded the preliminary step of our search but met the review inclusion criteria. In total, we included 40 qualifying studies, with a final population of 47 patients, in our analysis (Figure 3) [7–46]. The patients' general and clinical characteristics are summarized in Table 1. Mean maternal age was 28,66 years (range 19-47). There was absence of comorbidity in 24/47 patients; instead 21/47 cases presented diseases related to development of PRES and 2/47 cases had comorbidity not linked with PRES. The onset of the disease regarded early puerperium in 13/47 cases and late puerperium in 34/47 cases. Seizures were revealed in 39/47 cases. Forty-five patients reported other symptoms. Instrumental diagnosis was obtained only by CT in 2/47, only by MRI in 25/47, by CT and MRI in 19/47, and by CT, MRI, and CTA in 1/47 patients. For what concern medical treatment 9/47 patients were treated only with antiepileptic prophylactic or therapeutic drug (magnesium sulfate, benzodiazepines, gardenale, levetiracetam, and valproate), 4/47 only with antihypertensive drug (calcium channel blockers, angiotensin receptor blockers, nitroderivates, beta receptors blockers, and diuretics), 23/47 with a combined antiepileptic and antihypertensive therapy, and 10/47 receiving a multidrug treatment including additional drugs (such as steroids, Acetylsalicylic Acid, Low Molecular Weight Heparin, Propofol, Paracetamol, and Codeine); finally one patient was treated with multidrug therapy associated with Plasma Exchange. Mechanic ventilation was necessary in 40/47 cases and 19/47 patients needed to admission in Intensive Care Unit (ICU). Early onset complications occurred in 9/47 cases; meanwhile only 2/47 cases reported long-time complications. One patient died and 44/47 showed a full remission. Mean time to clinical remission was 10,69 days (range 2-45) (Table 2).

## 5. Discussion

Posterior reversible encephalopathy syndrome (PRES) is a rare disorder associated with acute hypertension; its exact incidence remains unknown. The pathogenesis of PRES is not clear; it seems to be associated with a rapid development of hypertension that leads to a malfunction of cerebral autoregulation; in particular in occipital lobe where the sympathetic innervation is less widespread, resulting in focal vasogenic edema [47–50]. Other conditions related to PRES are also chemotherapy, infection, sepsis, autoimmune diseases, and hypercalcemia (cytotoxic edema). Indeed, a leading hypothesis suggests a crucial role for endothelial dysfunction and activation in PRES pathogenesis [1]. PRES is characterized by transient neurologic signs including headache, visual changes, seizures, and altered sensorium [14]. Cortical blindness is considered a typical and characteristic symptom of this syndrome [18]. PRES is reversible in a few days but if appropriate management is delayed there is high risk of permanent neurologic damage secondary to cerebral infarction or hemorrhage and transtentorial herniation resulting in death [47]. Subjective cognitive problems, development of chronic epilepsy, and progress to irreversible (partial) blindness can be long-time consequences after years from acute episode [51]. Early and late complication such as pulmonary edema, dissection of extracranial internal left carotid artery, cerebral herniation, short term memory loss, subarachnoid hemorrhage, permanent mild dysmetria, visual impairment, and death have been described [9, 19, 24, 36]. Early recognizing of symptoms is fundamental for a timely diagnosis. As reported in literature cerebral MRI is the gold standard diagnostic tool; neuroimaging performed shows diffuse edema of the white matter, which selectively involves the parietooccipital regions of the brain; edema usually shows iso- or hypointensity in DWI [49]. Lee et al. reported a study with 136 cases of PRES including patient unrelated to pregnancy. MRI performed in these patients showed vasogenic edema localized in the occipital and parietal lobes (98%), but also in frontal lobe (68%), temporal lobe (60%), cerebellum (32%), and basal ganglia (14%) [47]. The initial evaluation of patients with PRES should focus on a rapid correction of blood pressure, hydration using crystalloid fluids, and maintenance of adequate oxygenation [14]. Pande et al. stated that PRES due to eclampsia showed a better prognosis than PRES caused by other risk factors [48]. Liman et al. compared 24 patients with preeclampsia-eclampsia associated PRES and 72 patients with PRES of other predisposing causes and in the first group showed frequent complete resolution of edema and less frequent residual structural lesions [51]. Demirel et al. suggested that timely supplementation of thiopental infusion to antihypertensive and magnesium sulfate treatment can improve the clinical status faster and more efficiently in patients with PRES to avoid persistent damage [52]. We reported three cases of PRES developed during puerperium, in which timely recognizing of patient's symptoms reached us to perform an early diagnosis and sudden therapy. In case of patients with a postpartum diagnosis of PRES, early intervention focused on monitoring vital parameters and MRI images and a treatment focused on hypertension control; cerebral edema reduction is a successful therapy which allowed us to avoid neurological sequelae, early and late complications, and patient's death. Performing a cerebral MRI in the suspicious of PRES clinicians should be aware to detect signs of cytotoxic edema that is a sign of the development of the disease [5, 49, 53]. The spread and the localization of edema are variable and could depend on the latency time between the seizure and the MRI. There is a large variability also in time of cerebral MRI normalization. According to the literature, despite the importance of cytotoxic edema, it is not linked to poor prognosis or to the development of early or late sequelae. In the analysis of cerebral lesion and in order to obtain a right diagnosis it is useful perform an accurate MRI examination using Apparent Diffusion Coefficient (ADC) maps and Diffusion Weighted Imaging. Despite an increased signal intensity in ADC maps, it is considered indispensable to differentiate vasogenic edema from cytotoxic edema in patients with PRES; Diffusion Weighted Imaging is more sensitive for detecting ischemic lesions and cytotoxic edema than are ADC maps [54]. Conversely, the positivity of ADC evaluates the reversibility of the damage by expressing the vasogenic edema [55]. Restricted diffusion is a typical finding in PRES as cytotoxic edema is not necessarily liked to irreversibility or to development of sequelae [55]. An ulterior MRI pattern to evaluate is the presence of an increased leptomeningeal enhancement in Fluid Attenuated Inverse Recovery (FLAIR) sequence in these patients [56]. Agarwal e al. analyzed MRI imaging in 20 patients suffering from PRES and they found an increasing leptomeningeal enhancement in 35% of these patients. This is normally associated with other radiological findings of PRES but rarely is an isolated finding. The increased leptomeningeal enhancement is the result of an endothelial injury and an increase in microvascular permeability [56]. Our data analysis showed the presence of leptomeningeal enhancement in FLAIR sequence in only 1/3 case, whereas Gao et al. stated that most patients do not show any abnormal enhancement on postcontrast T1WI; it has been reported to occur in 21%–38% of patients with PRES according to the literature [57]. As regards EEG reports in patients suffering from PRES, it is important to note that numerous studies have focused on radiological or clinical findings of PRES; meanwhile EEG patterns are poorly described. Kastrup et al. retrospectively analyzed 49 patients affected by PRES and characterized epileptic focus activity in these patients in particular at frontal or occipital lobe [58]. In our case series one patient developed a combined frontal-occipital bifocal epileptiform activity, another an isolated occipital activity, and the last a peculiar left hemispheres epileptiform activity. No patient developed secondary epilepsy.

Nowadays the hypothesis of endothelial dysfunction in the pathophysiology of PRES is also proposed. For this reason monitoring LDH serum level as marker of endothelial dysfunction could be useful [59]. It is mandatory to remember that there are many severe obstetric complications that could be caused by endothelial dysfunction as preeclampsia, and so in these patients an isolated monitoring of LDH is not recommended, but a full screening for serum marker of preeclampsia. We revealed an increasing in two of three cases of PRES: in one patient the elevated LDH level is associated with thrombocytopenia, elevated liver enzymes, and increasing in markers of hemolysis and first depended on the developing of HELLP syndrome in a preeclamptic woman; meanwhile the other patients showed an isolated increasing in LDH level that could be linked to the developing of PRES, as reported in other cases in literature [59]. Approaching a woman suffering from headache after CS or a VD with intrapartum epidural a close monitoring is necessary in order to have a quick intervention in case of development of PRES.

## 6. Conclusion

PRES syndrome should always be considered in women with acute hypertension disorders associated with epileptic seizures or other neurological symptoms during pregnancy and in the postpartum. In our cases the patient obtained a complete remission of symptoms due to the early diagnosis and the sudden therapy. Our review stated the necessity to perform an instrumental diagnosis, using MRI as diagnostic gold standard tool and an adequate pharmacological and life support therapy in order to avoid any delay in diagnosis and treatment that may results in death or in irreversible neurological sequelae.

## Figures and Tables

**Table 1:** Review of the cases included in the literature.

**N**	**Authors**	**Age**	**Onset (Puerperium)**	**Instrumental Diagnosis**	**Seizures**	**Other symptoms** **CNS not CNS**	**Treatment**	**MV**	**Early complications **	**ICU **	**Results** ** Follow Up** **MRI**	**Clinical Outcome**
1	Cozzolino M. [7]	32	Early	CT, MRI	No	Yes Yes	Antiepileptic Antihypertensive	No	No	Yes	Normal	Full remission

2	Zis P. [8]	35	Late	CT, MRI	Yes	Yes No	Antiepileptic	Yes	No	No	Normal	Full remission

3	Orehek E. [9]	26	Late	CT, MRI	Yes	Yes No	Antiepileptic Antihypertensive	Yes	Cerebral Herniation	Yes		Mild left arm dysmetria and persistence of brisk muscle strecht reflexes

4	Kauntia R. [10]	27	Late	MRI	No	Yes No	Antihypertensive	No	No	No	Normal	Full remission

5	Aygun B.K. [11]	23	Early	MRI	No	Yes No	Multi drug	No	No	No	Normal	Full remission

6	Peng W.X. [12]	36	Early	MRI	Yes	Yes No	Multi drug	No	No	No	Normal	Full remission

7	Pizon A.F. [13]	27	Late	MRI	Yes	Yes Yes	Antiepileptic Antihypertensive	No	No	Yes		Full remission

8	Servillo G. [14]	27	Late	MRI	Yes	No No	Antiepileptic Antihypertensive	Yes	No	Yes	Normal	Full remission

9	Servillo G. [14]	24	Early	MRI	Yes	Yes No	Antiepileptic Antihypertensive	No	No	Yes	Normal	Full remission

10	Servillo G. [14]	29	Late	MRI	No	Yes No	Antiepileptic Antihypertensive	No	No	Yes	Normal	Full remission

11	Servillo G. [14]	27	Late	MRI	Yes	Yes No	Antiepileptic Antihypertensive	No	Subarachnoid Hemorrhage	Yes	Death	Death

12	Patil V.S. [15]	21	Late	CT, MRI	Yes	Yes No	Antiepileptic Antihypertensive	No	No	No	Normal	Full remission

13	Maggi G. [16]	35	Early	CT, MRI	Yes	Yes No	Antiepileptic Antihypertensive	No	No	Yes	Normal	Full remission

14	Babahabib M.A. [17]	31	Early	MRI	Yes	Yes No	Multi drug	No	No	Yes	Normal	Full remission

15	Doherty H. [18]	19	Late	MRI	Yes	Yes No	Antiepileptic	No	No	No	Normal	Full remission

16	Gimovsky M.L. [19]	25	Late	CT, MRI	Yes	Yes No	Multidrug	No	Short-Term Memory Loss Lupus Cerebral Vasculitis	Yes	Normal	Full remission

17	Papoutsis D. [20]	27	Late	CT, MRI	Yes	Yes No	Antiepileptic Antihypertensive	Yes	No	Yes	Normal	Full remission

18	Ehtisham S. [21]	30	Late	MRI	Yes	Yes Yes	Antihypertensive	No	No	Yes	Normal	Full remission

19	Gomez-Gonzales C. [22]	38	Early	MRI	Yes	Yes No	Antiepileptic	No	No	Yes	Normal	Full remission

20	Kameda G.W. [23]	30	Late	MRI	Yes	No No	Antiepileptic Antihypertensive	Yes	No	Yes	Normal	Full remission

21	Lawson G. [24]	47	Late	MRI	No	Yes No	Antiepileptic Antihypertensive	No	Partial Scotoma	No		Mild visual blurring at watching television

22	Lemmens R. [25]	30	Late	MRI	Yes	Yes No	Multi drug	No	Loss of consciousness for two days	No	Normal	Full remission

23	Negro A. [26]	37	Early	MRI	Yes	Yes No	Multidrug Plasma Exchange	No	No	No	Normal	Full remission

24	Pezzi M. [27]	35	Late	MRI	Yes	Yes No	Antiepileptic Antihypertensive	No	No	Yes	Normal	Full remission

25	Siddiqui T.S. [28]	35	Late	MRI	Yes	Yes No	Antiepileptic Antihypertensive	No	No	Yes	Normal	Full remission

26	Singhal A.B. [29]	21	Late	CT, MRI, TCA	Yes	Yes No	Antiepileptic Antihypertensive	No	Minor subarachnoid hemorrhage	No	Normal	Full remission

27	Singhal A.B. [29]	23	Late	CT, MRI	Yes	Yes No	Antiepileptic Antihypertensive	No	No	No	Normal	Full remission

28	Singhal A.B. [29]	31	Late	MRI	Yes	Yes No	Antiepileptic Antihypertensive	No	Dissection of ELICA	No	Normal	Full remission

29	Uwatoko T. [30]	30	Late	CT, MRI	No	Yes No	Multi drug	No	No	No	Normal	Full remission

30	Wahab W. [31]	20	Late	CT	Yes	Yes No	Antihypertensive	No	No	No	Normal	Full remission

31	Wernet A. [32]	24	Early	CT, MRI	Yes	Yes No	Antiepileptic Antihypertensive	No	No	No	Normal	Full remission

32	Zhang M. [33]	27	Late	MRI	Yes	Yes No	Multi drug	No	No	No	Normal	Full remission

33	Etesse B. [34]	23	Early	MRI	Yes	Yes No	Antiepileptic	No	No	No	Normal	Full remission

34	Farissier F. [35]	35	Late	CT, MRI	No	Yes No	Multi drug	No	No	No	Normal	Full remission

35	Bakkali H. [36]	23	Late	CT, MRI	Yes	Yes Yes	Antiepileptic Antihypertensive	Yes	Acute pulmonary edema	No	Normal	Full remission

36	Finocchi V. [37]	28	Late	CT, MRI	Yes	Yes No	Antiepileptic	No	No	No	Normal	Full remission

37	Finocchi V. [37]	30	Late	CT, MRI	Yes	Yes No	Antiepileptic	No	No	No	Normal	Full remission

38	Finocchi V. [37]	30	Late	CT, MRI	Yes	Yes No	Antiepileptic	No	No	No	Normal	Full remission

39	Cho H.J. [38]	31	Late	CT, MRI	Yes	Yes No	Antiepileptic Antihypertensive	No	Short-term memory loss Pulmonary edema	No	Normal	Full remission

40	Onrubia X. [39]	23	Early	CT, MRI	No	Yes Yes	Antiepileptic Antihypertensive	No	No	Yes	Normal	Full remission

41	Tsukimori K. [40]	28	Early	MRI	Yes	Yes No	Multidrug	No	No	No	Normal	Full remission

42	Prout R. [41]	32	Late	CT, MRI	Yes	Yes No	Multi drug	No	No	Yes	Normal	Full remission

43	Torrillo T.M. [42]	32	Late	MRI	Yes	Yes No	Antiepileptic	No	No	No	Normal	Full remission

44	Chiu-Ming H. [43]	33	Late	MRI	Yes	Yes No	Antiepileptic	No	No	No	Normal	Full remission

45	Domingues-fuentes B. [44]	25	Late	CT, MRI	Yes	Yes No	Antiepileptic Antihypertensive	Yes	No	Yes	Normal	Full remission

46	Oyinloye O.I. [45]	20	Early	CT	Yes	Yes No	Antiepileptic Antihypertensive	No	No	No	Normal	Full remission

47	Garg R.K. [46]	25	Late	MRI	Yes	Yes No	Antihypertensive	No	No	No	Normal	Full remission

CNS: central nervous system, MV: mechanic ventilation, ICU: intensive care unit, MRI: magnetic resonance imaging, CT: computed tomography, CTA: computed tomographic angiographic; Multidrug: therapy including antiepileptic, antihypertensive, and other kind of drugs such as diuretics or antiplatelets or anticoagulants, and ELICA: extracranial internal left carotid artery.

**Table 2:** Summary of patients' characteristics, clinical data, and outcome.

**Variable**	**N of patients (**%**)∗**
Mean Maternal Age (years), range	28,66 (19-47)

Comorbidity	
(i) Absence	24 (51%)
(ii) Related with development of PRES	21 (45%)
(iii) Not related with development of PRES	2 (4%)

Onset	
(i) Early puerperium	13 (28%)
(ii) Late puerperium	34 (72%)

Instrumental Diagnosis	
(i) CT	2 (4%)
(ii) MRI	25 (54%)
(iii) CT, MRI	19 (40%)
(iv) CT, MRI, CTA	1 (2%)

Seizures	
(i) Absence	8 (17%)
(ii) Presence	39 (83%)

Other symptoms	
(i) Absence	2 (4%)
(ii) Isolated headache	7 (15%)
(iii) Other neurological symptoms	29 (62%)
(iv) Association of neurological/not neurological symptoms	4 (9%)

Therapy	
(i) Antiepileptic treatment	9 (19%)
(ii) Antihypertensive treatment	4 (9%)
(iii) Antiepileptic + Antihypertensive treatment	23 (49%)
(iv) Multi drug therapy	10 (21%)
(v) Multi drug therapy + Plasma Exchange	1 (2%)

Mechanic Ventilation	
(i) Performed	40 (85%)
(ii) Not Performed	7 (15%)

ICU admission	
(i) Necessary	19 (40%)
(ii) Unnecessary	28 (60%)

Early onset complications	
(i) Absence	38 (81%)
(ii) Nervous Central System	5 (11%)
(iii) Cardio-Pulmonary System	2 (4%)
(iv) Multi-organ complications	2 (4%)

Clinical outcome	
(i) Death	1 (2%)
(ii) Full remission	44 (94%)
(iii) Presence of long time complications	2 (4%)
(iv) Mean time to remission (days), range	10,69 (2-45)

Time to instrumental follow up (days)	
(i) Mean time, range	38,05 (5-365)

*∗*Unless otherwise specified.

CT: computed tomography, MRI: magnetic resonance imaging, and CTA: computed tomographic angiographic.
